# Correlation between the Spread of COVID-19 and the Interest in Personal Protective Measures in Poland and Portugal

**DOI:** 10.3390/healthcare8030203

**Published:** 2020-07-09

**Authors:** Artur Strzelecki, Ana Azevedo, Alexandra Albuquerque

**Affiliations:** 1 Department of Informatics, University of Economics in Katowice, 40-287 Katowice, Poland; 2 CEOS.PP, Porto Accounting and Business School, Polytechnic Institute of Porto, 4200-465 Porto, Portugal; aazevedo@iscap.ipp.pt (A.A.); alexalb@iscap.ipp.pt (A.A.)

**Keywords:** COVID-19, personal protective equipment, face mask, sanitizers, hand hygiene

## Abstract

The pandemic of the coronavirus disease 2019 (COVID-19), has gained extensive coverage in public media and global news, generated international and national communication campaigns to educate the communities worldwide and raised the attention of everyone. The coronavirus has caused viral pneumonia in tens of thousands of people around the world, and the COVID-19 outbreak changed most countries’ routines and concerns and transformed social behaviour. This study explores the potential use of Google Trends (GT) in monitoring interest in the COVID-19 outbreak and, specifically, in personal protective equipment and hand hygiene, since these have been promoted by official health care bodies as two of the most protective measures. GT was chosen as a source of reverse engineering data, given the interest in the topic and the novelty of the research. Current data on COVID-19 are retrieved from GT using keywords in two languages—Portuguese and Polish. The geographical settings for GT are two countries: Poland and Portugal. The period under analysis is 20 January 2020, when the first cases outside China were known, to 15 June 2020. The results show that there is a correlation between the spread of COVID-19 and the search for personal protective equipment and hand hygiene and that GT can help, to a certain extent, understand people’s concerns, behaviour and reactions to sanitary problems and protection recommendations.

## 1. Introduction

At 10 am (CET) on 15 June 2020, over 8 million cases of severe acute respiratory illness caused by the novel coronavirus (2019-nCOV), known as COVID-19, have been reported globally. Out of those, 431,541 cases have been lethal and the disease has hit 195 countries and territories [1,2]. It took over three months to reach the first 100,000 confirmed cases, 28 days to climb above 1 million and only 13 days to reach 2 million confirmed cases [1]. The first infected cases in late 2019 are connected to the Wuhan seafood market in China [3] and strong similarity with other previously well-known respiratory diseases, such as Severe Acute Respiratory Syndrome (SARS-CoV) and the Middle East Respiratory Syndrome–Coronavirus (MERS-CoV) [4] was observed. As at 10 am (CST) on 22 January 2020, Wuhan international airport was closed due to the threat of international dissemination of this disease via commercial air travel [5]. Other public transportation in the city was also suspended. In just a few weeks the disease spread to a large number of other countries, and on 30 January the World Health Organization (WHO) Director-General declared the coronavirus outbreak a public health emergency of international concern. On 11 February the official name of COVID-19 was announced for the virus responsible for the new respiratory disease (previously known as “2019 novel coronavirus” or “2019-nCoV”) [6]. The coronavirus belongs to a large family of viruses that can cause many different infections, from a cold to acute respiratory failure syndrome [7]. The one from Wuhan is only the 7th that is known to harm people [8] and to the time being there is no single effective way to fight coronaviruses, but only measures to help relieve symptoms, the most common including fever, respiratory problems and lung infiltration [9].

According to Wörl, R. et al. [10] “from the current point of view, SARS-CoV-2 is transmitted mainly by droplet infection. The incubation period is assumed to be a maximum of 14 days. In addition to direct contact with an infected person, likely, the infection can also occur via hand or surface contact (smear infection)”. Therefore, general protection measures include performing hand hygiene frequently with an alcohol-based hand rub; avoiding touching eyes, nose and mouth; practising respiratory hygiene; wearing a medical mask if having respiratory symptoms, keeping physical and social distance, among others [11]. Since news media and official sources were instantly reporting the current number of infected and new cases in new countries and territories [12,13], and measures across countries, like contingency plans and information on protection measures, have been rousingly demanded by governments, people started to be concerned with their health, due to coronavirus strong and fast *transmission speed* [14,15]. This triggered global interest in personal protection equipment (PPE) [16]. PPE like face masks, gloves, gowns and goggles is considered effective against COVID-19 when used in health and medical contexts [17]. Also, public interest was shown on face masks and sanitizers [18], most likely because the first images from protection measures came from China, where the use of face masks in a possible infection scenario is seen as safer and more considerate, the reason why some of the Asian governments were urging to the massive wearing of masks. However, the WHO started by discouraging the use of mask unless by persons with respiratory symptoms and by health staff and considered that the global demand on PPE was driven not only by the number of COVID-19 cases but also by misinformation, panic buying and stockpiling [19]. Respiratory etiquette and hand hygiene have been, on the other hand, very encouraged by WHO since the beginning of the outbreak as more effective personal protection measures.

There is little doubt that people all over the world express a personal interest in this topic by searching for information and using search queries. Currently, rapid reports on the use of search data refer to searches in “handwashing” [20] or “wash hands” [21], “loss of smell” [22], “other symptoms” [23] of COVID-19 or correlation of coronavirus to “SARS” and “MERS” [24]. However, there is little knowledge if the interest in PPE and hand hygiene is different or the same in different countries. The motivation behind this study is, therefore, to use Google Trends (GT) data to analyze how the search for information about face mask and hand hygiene, during the coronavirus outbreak, is reported in two European countries: Poland and Portugal.

The study aims to explore the correlation of the spread of COVID-19 and the interest in Personal Protective Measures (PPM), like face masks and sanitizers. By measuring this correlation, it is possible to make nowcast, policy implications and other suggestions to state parties in terms of taking additional health measures, namely concerning information and advice for the public.

The paper is organized as follows. Section 2 includes the method, GT concept and material for data retrieval and processing. Section 3 refers to the results, while Section 4 presents the discussion. In the last section, authors highlight the contribution of the research, discuss its limitations and draw conclusions about the results.

## 2. Materials and Methods 

An area of scientific research that focuses on scanning the Internet, publicly available data and other sources for user-contributed health-related content is named infodemiology [25]. In recent years, a lot of research has been done using data collected from Google Flu Trends or Google Health API. Recently there has been a growing number of research studies using GT [26,27]. Before its release, early studies were based on Google Flu Trends, a source for queries connected to diseases [28]. GT is the source of reversed engineered data. It shows what was searched in Google, normalizes the data in terms of search frequency and presents it in relative search volumes. Data are segmented into years, months, days, and geographical regions. Researchers can compare a maximum of 5 keywords or topics using segments in one try. Studies on GT are mainly used to examine the seasonality [29] and can be divided into four areas: infectious diseases, mental health, other diseases and general population behaviour [30].

The methodology used in this study is based on [31] in terms of data collection and on [32] in terms of data analysis. Data for this research are collected from GT (https://trends.google.com/trends) and is normalized. The highest interest on search query is expressed by 100, whereas lack of interest or insufficient data are expressed by 0. GT contains data from different geographical locations, segmented into countries, territories and cities and also allows to set the custom time range. Queries are collected from five specialized search engines: Web, Image, News, Google Shopping and YouTube Search. Data in GT are an anonymized, unfiltered sample of actual search requests made in Google, which makes it impossible to determine whether searches are made by individuals or companies or the exact intention of the requested search. However, data are categorized into topics, determined by the search query, which allows processing the interest in a particular topic in different countries. Our sample selection process was based on four parameters: countries, period, new cases and keywords. The countries were selected as a convenient sample that could be the subject of the study: the home countries of the authors. This parameter was, therefore, incredibly prompt, uncomplicated, and economical. Data were collected from Web Search with two geographical settings: (1) Portugal, to see the interest in PPM in Portuguese; (2) Poland, to see the interest in PPM in Polish.

The period was defined between the first reported cases of the disease outside China mainland (20 January 2020) [2] and 15 June 2020. As far as the third parameter is concerned—new cases—we have used data in line with European Centre for Disease Prevention and Control (ECDC)’s copyright policy. The downloadable data file is updated daily and contains the latest available public data on COVID-19 [33]. Data are segmented into days, month, year, cases, death and countries. Finally, keywords were selected related to PPM in Portugal and Poland, as described in the following section.

### 2.1. Keyword Selection

According to Mavragani & Ochoa [31], the terms used for the Google trends analysis are of paramount importance, to be able to get reliable results. For this reason, and based on the topic of this paper—personal protective measures against the new coronavirus—keywords related to PPE and hand hygiene were both looked into in Portuguese and Polish. In Portuguese, the reference terminology was from “Plano Nacional de Preparação e Resposta à Doença por Novo Coronavírus (COVID-19)” [34]. The selected terms were *máscara cirúrgica* (face mask), *desinfetante* (sanitizer) and *álcool* (alcohol). The full list of terms related to PPE includes gloves, face masks, respirators, goggles, aprons, face shields, gowns, shoe protection and hair caps. However, the most referred to equipment in mass and social media over the time covered by this paper was face mask. Moreover, the other PPE terms were tested in GT and the hits were irrelevant.

Face masks were and still are, when available, one of the most bought protective items in Portugal, running almost completely out of stock all over the country, especially after the outbreak in Italy, by Carnival [35]. However, the term mask (*máscara*) in Portuguese is used in several domains and contexts, which could raise some false hits in GT. First of all, in the health domain only, we can find different designations for mask (*máscara*) apart from mask alone, such as, for instance, *máscara de proteção* (protective mask) or *máscara cirúrgica* (face mask, surgical mask). Secondly, because a mask is also a Carnival adornment, which in 2020 took place on 25 February, just about when the coronavirus outbreak was being known in Italy. Finally, one of the Portuguese TV Channels was broadcasting a very popular talent show called “A Máscara”, based on the American talent show “The Masked Singer”, and the season finale took place on 23 February.

Therefore, when we searched for *máscara* in Google Trends, on 10 March, we could find that some of the related queries mentioned Carnival and the TV show. For this reason, and in order to have more accurate results, we decided to use a narrower term, *máscara cirúrgica* (face mask), since this is also the designation found in the face mask packages. As far as hand hygiene is concerned, we decided to use the broader term *desinfetante* (sanitizer), instead of *desinfetante para mãos* (hand sanitizer) or *gel desinfetante* (sanitizer gel), because all related terms in GT were concerned with *desinfetante para as mãos* (hand sanitizer). *Álcool*, in Portuguese, as in other languages, is not only a term used to designate an antiseptic substance but also a spirit. However, all the related keywords in our GT search were only related to the antiseptic meaning of the term. After preliminary research in the Polish language, we have chosen the following keywords to examine: *maseczki* (face masks), *dezynfekcji* (sanitizer) and *antybakteryjny* (antibacterial). Contrarily to the semantic challenges in Portuguese, there were no overlapping meanings or other related keywords in Polish, which allowed us to select the search terms in a much more straightforward way. Data used for analysis are generated in a single enquiry so that keywords’ scores in GT are adjusted. This method seemed to allow us to observe the volume of interest between the keyword searches.

### 2.2. Data Analysis

Data analysis started with the use of visualization [36] to understand the trends of the data related to the various keywords. Line graphs including the variations of the searches in GT of the three keywords, and the number of new cases for each of the countries, were used in a first approach. The trends found out through the visualization of the graphs were summarized. In addition, the numerical description of the data was included, summarizing the distributions by the use of measures of central tendency (mean, minimum, 1st quartile, median, 3rd quartile, and maximum), and measures of dispersion (standard deviation, range, and interquartile ranges). Box Plot Diagrams were used to visualize the distribution of the data. The data analysis for this paper was generated using MsExcel^TM^ 2016 with support of the add-in “Real Statistics Resource Pack software (Release 6.8), Copyright (2013–2020), Charles Zaiontz (www.real-statistics.com).”

To be sure if the differences found in the keywords’ trends in both countries were statistically significant, a t-student test has been applied for two paired samples [37,38], with formulas of MsExcel^TM^ 2016. 

The test was applied for the null hypothesis. H_0_, against the alternative hypothesis, H_1_, to each of the keywords:

***H*_0_.** (*maseczki, máscara cirúrgica*): $\mu 1_{PT}$ = $\mu 1_{PL}$

***H*_1_.** (*maseczki*, *máscara cirúrgica*): $\mu 1_{PT}$ ≠ $\mu 1_{PL}$

***H*_0_.** (*dezynfekcji*, *desinfetante*): $\mu 2_{PT}$ = $\mu 2_{PL}$

***H*_1_.** (*dezynfekcji*, *desinfetante*): $\mu 2_{PT}\neq$ $\mu 2_{PL}$

***H*_0_.** (*antybakteryjny*, *álcool*): $\mu 3_{PT}$ = $\mu 3_{PL}$

***H*_1_.** (*antybakteryjny, álcool*): $\mu 3_{PT}\neq$ $\mu 3_{PL}$

In light of this, it seemed important to identify groups of days with similar searches. Moreover, data were analyzed using machine learning techniques to obtain groups of days with similar searches for Portugal and Poland, separately. Clustering was used, since “Clustering allows the identification of homogeneous groups containing several elements which have high similarity with all the other elements of the same cluster, and that have low similarity to all the elements of the other groups” [39]. The k-means algorithm was selected as the clustering algorithm. Despite being accepted as a simple and efficient algorithm for clustering, one issue related to the use of the k-means algorithm is that the ideal number of clusters is not predefined by the algorithm, being determined by the use of some accepted heuristics [40,41,42,43]. Several experiments have been developed with a gradual increase in the number of clusters and the corresponding analysis of the obtained errors have been performed. However, in light of the poor analysis’ results as well as the relatively small size of the dataset, the authors decided to consider only two clusters. Additionally, RapidMiner Studio version 9.7 (RapidMiner GmbH, Dortmund, Germany) was used and two similar RapidMiner Studio processes were implemented, one for each country.

Finally, data were analyzed in search for correlations [37] between the keywords and the number of new cases in Poland and Portugal respectively. This was done because this study aims to understand if the spread of COVID-19 is related (i.e., correlates) with the interest in PPM. The spread of COVID-19 is represented by the variable new cases, whilst the interest in PPM is represented by each of the GT searches for the keywords. Firstly, an intuitive approach was used by constructing scatterplots with the new cases variable in the X-axe and each of the keywords searches in the Y-axe, adjusting a straight line which shows the existence of a linear relationship. Following, the Pearson Correlation Coefficient was obtained. This coefficient varies from −1 to +1. Disregarding the signal, the closer the coefficient value is to 1, the stronger the relationship between the two variables. In order to verify if the Pearson Correlation Coefficient varies as time goes by, it was also calculated considering lags of 1 day to 10 days, both for Portugal and for Poland. The *p*-values for the tests were also obtained to confirm the statistical significance of the correlations. Both the scatterplots and the correlations were obtained using MsExcel^TM^ 2016.

## 3. Results

This section presents the results obtained with the data analysis, considering a 3-folded approach. Firstly, we present the general trends found in the data. Secondly, the groups of days with similar searches were identified. Finally, the results of the correlations between the spread of COVID-19 and the interest in the PPM are presented.

### 3.1. General Trends

**Figure 1:**
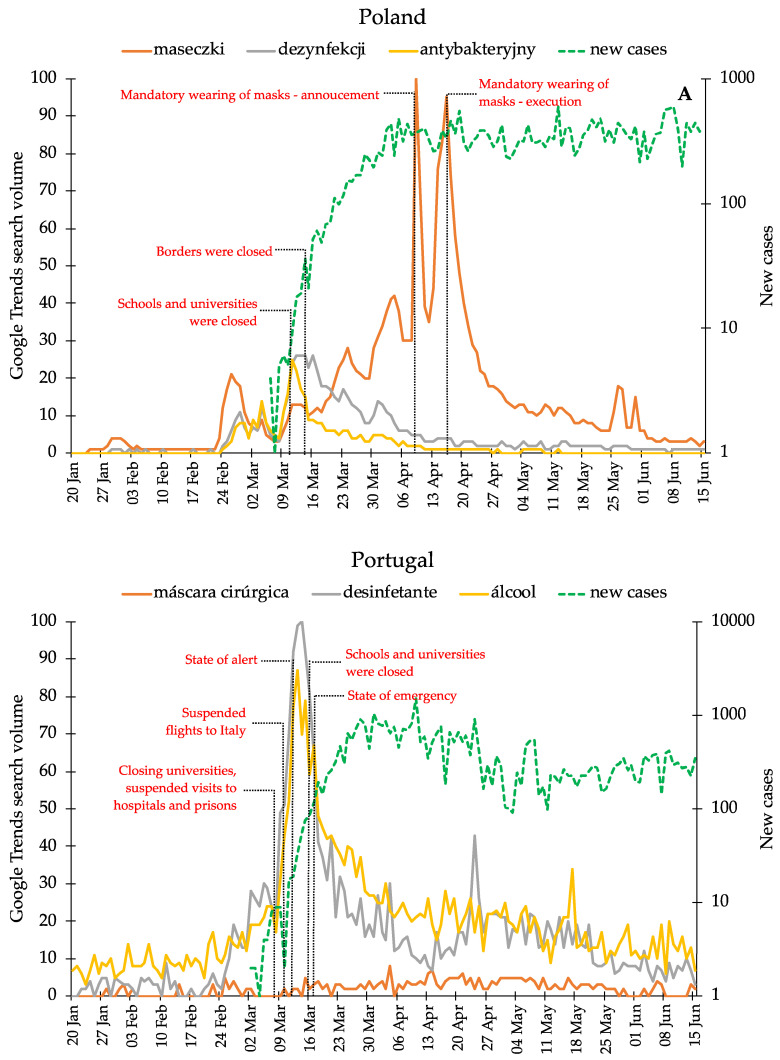
Evolution of the keywords in GT in Poland and in Portugal from 20 January to 15 June compared to the number of new cases in each country respectively.

**Figure 2:**
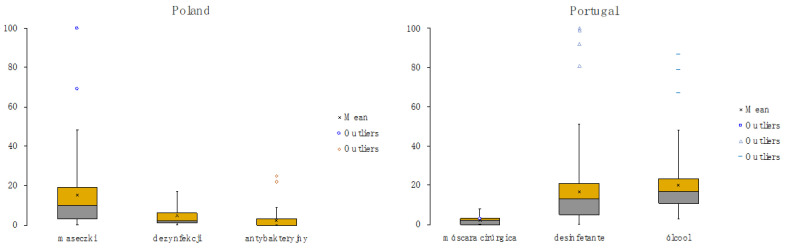
BoxPlot graphs of the keywords in GT in Poland and Portugal from 20 January to 15 June.

Figure 1 shows the evolution of the keywords searched in GT from 20 January to 15 June in Poland and Portugal. Table 1 presents a numerical description of the data including measures of central tendency, namely mean, minimum, 1st quartile, median, 3rd quartile, and maximum, and measures of dispersion, namely standard deviation, range, and interquartile ranges. Figure 2 presents the box plot diagrams for both countries. We can observe that there is a wide dispersion in the distributions, which present very high standard deviations, higher than the mean in almost all the cases. Following, a summary of the main trends that can be found in Figure 1 and Figure 2 is presented.

In Poland, the most searched keyword is *maseczki*, with a peak on 26 February. On 2 March the searches with the keywords *dezynfekcji* and *antybakteryjny* are higher than the searches with the keyword *maseczki*. The keyword *dezynfekcji* presents peaks on 12 March and 16 March, followed by a downward trend, except on 20 March when a higher hit than *maseczki* can be found. The keyword *antybakteryjny* presents a peak on 11 March, followed by a downward trend, except on 15 March when, again, it has a higher hit than *maseczki*. The keyword *maseczki* achieves a peak on 9 April, followed by a fall, tending to rise again on 16 April. Interestingly, the searches with all the keywords present an upward trend at the end of February and then again when the first confirmed cases emerge (between 7 and 9 March). The number of new cases stabilize at the beginning of April, after having suffered a significant rise during March.

In Portugal, Figure 1 shows that the interest in all the keywords is low until the rise of new cases at the beginning of March, when the searches with the three keywords, *máscara cirúrgica*, *desinfetante*, and *álcool* slightly increase. The most searched keyword is *álcool* until 25 February, when the searches with the keyword *desinfetante* have more hits. The peak of the keyword *desinfetante* is to be seen on 3 March and on 13 March, followed by a downward trend. The searches with the keyword *álcool* peaked on 13 March and on 17 March, also followed by a downtrend. Concerning the searches with the keyword *máscara cirúrgica*, it can be observed that they are not relevant in Portugal in the first period under analysis, but show an increasing trend at the beginning of April, similar to levels of *desinfetante* and *álcool*. The number of new cases stabilize at the end of March, after the considerable rise at the beginning of the month.

In sum, it can be observed that the two countries have different trends. It can be said that the interest in the keyword *maseczki* is deeper in Poland, revealing a big increase in April, while in Portugal the keywords *desinfetante* and *álcool* are more relevant, compared to the low hits of the keyword *máscara cirúrgica*.

Following, t-students tests have been applied to ascertain if the differences found in the trends of the two countries were statistically significant (cf. Table 2). We observe that the tests are statistically significant for all the three categories, thus we can reject the null hypothesis and conclude that there are differences in the use of all the three keywords between Poland and Portugal.

### 3.2. Group of Days with Similar Searches

Two datasets have been considered for the generation of the groups of days for each country, containing the keyword searches. 

As far as Poland is concerned, two groups of days were identified:

- cluster 1, comprising twenty-one days, from 1 April to 21 April
- cluster 0, all the other days of the period under analysis.

These results allow us to say that in Poland the considered period has similar types of searches, except for 1 April to 21 April, coincident with the high search volume (Figure 1). 

As far as Portugal is concerned, two groups of days were identified:

- cluster 1, comprising thirteen days, from 9 March to 21 March
- cluster 0, all the other days of the period under analysis.

In view of this, it can be said that in Portugal the considered period has similar types of searches, except between 9 March and 21 March, coincident with the high search volume for *desinfetante* and *álcool* (Figure 1).

Table 3 presents the values of the cluster centroids for both countries. It can be noticed that, concerning Poland, the main difference in cluster centroids is related to the value of the keyword *maseczki*, which is low for the cluster centroid 0, and high for the cluster centroid 1. Concerning Portugal, the main difference in cluster centroids is in the value of the keywords *desinfetante* and *álcool*, which is low for luster centroid 0, and high for cluster centroid 1. The results thus show that all days comprised by cluster 0 are closer to cluster centroid 0 than to the cluster centroid 1, and vice-versa, and that the centroids values validate the results emphasized above.

### 3.3. Correlations between the Spread of COVID-19 and the Keywords

**Figure 3:**
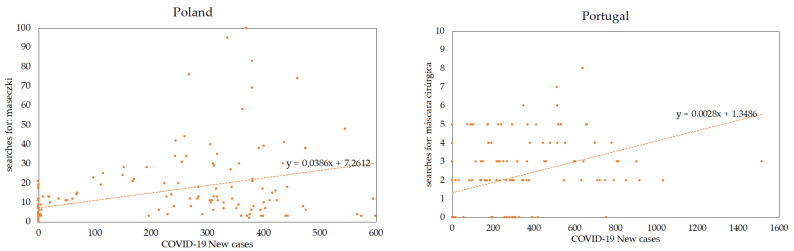
Scatterplots relating *new cases* with *maseczki* and *máscara* for Poland and Portugal, respectively.

To obtain a quick idea of the relation between the spread of COVID-19 and the interest in PPM, the scatterplots with the *new cases* variable in the X-axe and each of the keywords searches in the Y-axe can be found in Figure 3 below. A positive relation was only observed in the keywords *máscara*, in Portugal, and *maseczki,* in Poland, showing that the increase of new cases led to a higher search for these keywords. 

The Pearson Correlation Coefficients have been calculated between the series of searches of the keywords in Google Trends (GT) and the lagged series of the new cases of COVID-19, for each country, considering day lags from 0 to 10. 

Table 4 presents the evolution of the correlations between each keyword search volume in Poland and the number of new cases of coronavirus in the country. It is shown that only the keyword *maseczki* has a correlation (between 0.3 and 0.4) with the number of new cases and that this very strong correlation is to be observed for 10 days, despite slight variations. The other two keywords have similar correlations (between 0.2 and 0.4) with the number of new cases. Finally, *p*-values reveal very high statistical significance for all coefficients.

Table 5 presents the evolution of the correlations between the keywords’ search volume in Portugal and the number of new cases of coronavirus in the country. It can be noticed that the keyword *máscara cirúrgica* has a very strong correlation (between 0.5 and 0.6) with the number of new cases for 3, 6, 7, 8, 9 and 10-day lags, showing also strong correlations (between 0.4 and 0.5) with the other day lags. The other two keywords—*álcool* and *desinfetante*—have low correlations (below 0.2) with the number of new cases. Also, *p*-values reveal very high statistical significance for all coefficients. 

## 4. Discussion

The results show that there are correlations between the search for keywords and the evolution of new cases, which are identified in two-time clusters for each country. In this section, we will discuss the results within the context of each country, also taking into consideration contention measures and communication strategies.

We will discuss the results related to the keywords in two parts, first in Portugal and then in Poland.

Since the first images from the outbreak came from China, where, as explained before, the use of face masks is culturally normal in case of suspicion of infection and currently mandatory in many places [44], and being COVID-19 a respiratory disease, the purchase of masks was a normal reaction in both countries. These factors provoked the exponential rise of purchases of face masks, both in Portugal and Poland, precipitating a masks’ shortage, even for medical purposes [18], although surprisingly, in results, the keyword search volume was not very significant in Portugal. One of the reasons for this weak volume may have been the fact that the Portuguese keyword—*máscara cirúrgica* —had some limitations, as we explain in the following section. Additionally, wearing a mask is generally taken as a relatively well-known PPE, which may not demand much search for information online. Moreover, this low search volume is, till very recently in line with official recommendations by WHO, ECDS and Directorate-General for Health (DGS), for instance. Still today, there is no broad consensus among medical staff and national and international bodies if wearing face masks (e.g., surgical-type) outside of healthcare settings or by non-contaminated people during a pandemic offers effective protection or reduces transmission [45].

Therefore, the massive use of masks is still not recommended by ECDC, WHO, DGS, although since 6 April the official notices of those bodies have slightly changed, starting with WHO, stating that “potential advantages of the use of a mask by healthy people in the community setting include reducing potential exposure risk from an infected person during the “pre-symptomatic” period and stigmatization of individuals wearing a mask for source control.” [46] On the following days, ECDC [45] (8 April) and the DGS (13 April) [47] suggest that the use of a mask is a complementary protection measure, stressing, however, hand hygiene and social distance as more effective protection measures. Our results are in line with the communication flow from the international health bodies and DGS, adjusted to the severity of the pandemic situation in Portugal, since Figure 1 shows a slight increase in the search with the keyword *máscara cirúrgica* after 13 April. 

Most official public notices and information in Portugal have been stressing hand hygiene and social distance as more effective protection measures. Therefore, and contrarily to the results related to queries for mask, in Portugal, searches for hand hygiene-related information were relatively stable during the period under analysis. However, the results highlight that the groups of days of searches split in both countries. In Portugal, that can be observed from 9 to 21 March, when the search volume for *desinfetante* and *álcool* peaks, which corresponds to the findings of the group of days identified in Section 3.2, as belonging to cluster 1. Due to the increase of new cases (with a growth rate above 40% since the beginning of March), some public bodies and the Portuguese government started to implement the first contention measures on 8 March [48]. These contention and mitigation measures were intensified in the following days, and on 10 March Portugal suspends all flights to and from Italy, theatres, museums and libraries are closed in Lisbon and Porto, together with some public events and some universities and schools suspend in-presence classes. Also on 10 March, WHO General-Secretary states that “the threat of a pandemic has become very real” [49] and the next day the WHO announces COVID-19 outbreak a pandemic. Between 12 and 16 March, the Portuguese Government declares a list of new contention and mitigation measures, namely quarantine for epidemiological surveillance and all schools and universities have in-presence classes suspended till 9 April, which sent many public servers home for telework. On 17 March the President of the Portuguese Republic approved the State of Emergency, implemented by the Portuguese Government on 18 March. This period, 9–21 March, was, therefore, most likely when the Portuguese population was deeply aware of the severity of the sanitary problem of COVID-19. Moreover, it was also the period with the highest growth rates of new cases (with the highest rate so far—51%—on 14 March [50]). So, considering all this, PPM was at this time certainly also a higher concern and results appear to corroborate this, with the peak of the search for *desinfetante* and *álcool* in cluster 1, corresponding to the group of days between 9 and 21 March.

In Poland, results showed that groups of days of searches split, with a peak on 9 April. Poland implemented strong contention measures earlier than Portugal (at the beginning of March). However, on 9 April, the Polish government announced that the closure of educational institutions and international transport would continue till 26 April, the borders would remain “closed” until 3 May, and people entering Poland would still be required to be quarantined for 14 days [51]. A new control measure was planned to start from 16 April, making it obligatory to cover one’s nose and mouth in public places, with masks, scarves or any other form of face-covering [52]. It should be noticed that this announcement of the Polish government could partly explain the group of days from cluster 1, between 1 and 21 April, and the results of Section 3.2, where different types of searches could be observed. Contrary to Portugal, in Poland sanitizers were not “seen” and referred to as intensively in mass media or is official public notices. This can be a possible explanation for the late rise in the search of this keyword since people were still gathering information about necessary hand hygiene practices for personal protection while already lacking face masks. What is more, this is consistent with what has been found by Husnayain et al. (2020) [20] and Lin et al. (2020) [21].

Together, the present results confirm a correlation between the spread of COVID-19, the media news, health bodies’ recommendations, governmental measures, growth rate and social and commercial behaviour concerning personal protective measures, namely the wearing of masks and use of hand sanitizer. Despite the fact that there was some inconsistency in the keyword research and social and commercial behaviour of the two countries, namely as far as the search and purchase of masks is concerned in Portugal, for instance, results point that both countries seem to have had a different interest in PPM, accordingly their national health bodies’ position on the more effective measures to fight the spread of COVD-19. We could, therefore, see higher interest in sanitizers in Portugal, since this hand hygiene was officially considered a more effective protection measure contrarily to Poland’s higher interest in masks, earlier considered effective and made compulsory.

## 5. Conclusions

This exploratory research has presented search trends for Personal Protection Measures, in Poland and Portugal, in the early months of the spread of COVID-19 (from January 20 to June 15). After a thorough research, three keywords in each language have been selected, and their search trends analysed. Clustering to find periods of similar searches has been used and two clusters for each country have been identified. From the results, it can be concluded that the selected period and the number of searches for these keywords are statistically significant. Moreover, the correlation of selected keywords with the number of new cases reported in each country has been also analysed and, in both countries, the keyword *maseczki*/*máscara cirúrgica* shows a relation (i.e, positive correlations, as depicted in Figure 1, and Table 3 and Table 4) with *new cases*. This highlights a correlation between the spread of COVID-19 and the search for PPM, although with different interest levels in each measure.

### 5.1. Contribution

This exploratory study meant to explore the correlation of the spread of COVID-19 and the interest in PPM, like face masks and sanitizers. By measuring this correlation, we expected to assess the usefulness of GT in monitoring not only commercial behaviour, being therefore of particular interest in marketing purposes, but specifically collective social reactions to mass media and official notices and public communications in a context of sanitary emergency [53,54]. 

Among the many studies already available in various fields that use Google Trends data, many attempted to understand people or society through search activities and thereby predict behaviour and some with success [55]. Some of those studies focused on the influence of public opinion by mass media and in a context of elections, for instance, but the most common fields of knowledge using Google Trends have been medical, pharmaceutical, economics, and more recently politics and marketing, among others. However, still according to Le Nghiem et al. (2016) [55], the use of GT to index research on social phenomena, like communication, for instance, can be of high interest and a rising trend itself.

Given that our findings are based on a limited number of keywords and countries, the results obtained do not allow us to make generalizations. However, they have indicated that online social behaviour was in line with the official sources of public notices and information and that it changed when the information changed. Even if an exploratory and limited study, we believe that our results show that if the official bodies provide proper information during a disease outbreak this can, to a certain extent, preview and guide the population behaviour, as well as help better manage supplies during a pandemic. It could also decrease an excessive amount of information and misinformation circulating in affected countries which might cause public anxiety or panic. In response to the ongoing pandemic, our results demonstrated that GT could potentially define the proper timing and location for risk communication.

Notwithstanding the lack of similar research, we could compare our results with and further emphasise the relevance of the followed approach since the current pandemic is a new phenomenon, we believe this research has raised interesting questions concerning public communication in a pandemic context. Finally, although this specific study did not have a cultural approach as its main purpose, it raised some interesting issues that could be further and better explored. We, therefore, believe that this kind of comparative analysis, using GT, can certainly be of use for other fields, like, for instance, intercultural communication studies, namely health-related.

### 5.2. Limitations and Future Research Directions

These results must be interpreted with caution and a number of limitations should be borne in mind. First of all, the country comparison was merely opportunistic, due to the nationality of the researchers, and was not based on specific cultural features that could have highlighted more relevant results. Secondly, as far as research aims and objectives are concerned, this has to be considered an exploratory study that does not intend to provide conclusive answers but, mainly, show the importance of tools such as GT to research on how mass communication and especially public notice and information can influence the online search of the general audiences, in a pandemic context.

Nevertheless, also GT data have some limitations that cannot be overlooked. The first is that data about coronavirus, COVID-19, number of new cases, new restrictions in countries, among others, is changing rapidly every day. Thus results are only relevant on the reported date. Once data were retrieved using bilingual keywords, results were also dependent on the linguistic connotations and cultural context, which also interfered with the design of the search in GT, as it was the case of *máscara*. We believe that, had this study been developed in another timing, the results could have been different.

Another limitation concerns the sample size of personal protective measures. As we referred to above, PPM include much more measures than just PPE and hand hygiene. Also, PPE concerns a much broader list of equipment beyond face masks. These two types of measures and items in particular (face masks and sanitizers) were, however, the most massively referred to and discussed as PPM. Finally, face masks and sanitizers are also products (not only measures) and therefore also more suitable keywords to use in GT.

All these limitations are, however, new research opportunities, since social behaviour concerning PPM during the pandemic is dynamic and the knowledge concerning the wearing of PPE, hand and respiratory hygiene/cough etiquette and other PPM is increasing too. Therefore, now that general panic seems to have decreased in both countries, the focus seems to be more on getting out of the lockdown and that the search for masks seems to be decreasing, while other keywords like PPE are starting to be less unknown by the general public, there seems to be room to use Google Trends to study more correlations related to COVID-19. Another direction of future work could be the use of different GT methodology for cross-country comparison.

## Figures and Tables

**Table 1:** Descriptive numerical analysis of the dataset.

	Maseczki	Máscara Cirúrgica	Dezynfekcji	Desinfetante	Antybakteryjny	Álcool
	Poland	Portugal	Poland	Portugal	Poland	Portugal
Minimum	0	0	0	0	0	3
1st Quart.	3	0	1	5	0	10.75
Median	10	2	2	13	0	17
3rd Quart.	19	3	6	21	3	23.25
Maximum	48	8	17	51	9	48
SD	18.37	1.88	6.27	18.67	4.22	14.72
Range	100	8	26	100	25	84
Q1-Min	3	0	1	5	0	7.75
Med-Q1	7	2	1	8	0	6.25
Q3-Med	9	1	4	8	3	6.25
Max-Q3	81	5	20	79	22	63.75

**Table 2:** Summary of the t-student test for two paired samples for means.

Keywords	Means	Test Results	*p*-Value
*máscara cirúrgica*	$\mu 1_{PT}≃2.04$	≃9.01	$≃1.02\times 10^{-15}$
*maseczki*	$\mu 1_{PL}=15.02$	(>1.99, 2-tailed t-value)	(<α=0.05)
*desinfetante*	$\mu 2_{PT}≃16.74$	≃−10.84	$≃1.75\times 10^{-20}$
*dezynfekcji*	$\mu 2_{PL}≃4.65$	(<−1.99, 2-tailed t-value)	(<α=0.05)
*álcool*	$\mu 3_{PT}≃20.19$	−18.31	$≃9.27\times 10^{-40}$
*antybakteryjny*	$\mu 3_{PL}≃2.31$	(<−1.99, 2-tailed t-value)	(<α=0.05)

**Table 3:** Information about the centroids of the clusters for Poland and Portugal.

Poland			Portugal		
Keyword	Cluster 0	Cluster 1	Keyword	Cluster 0	Cluster 1
*maseczki*	9.500	54.889	*máscara cirúrgica*	2.059	1.833
*dezynfekcji*	4.577	5.167	*desinfetante*	12.206	68.167
*antybakteryjny*	2.377	1.833	*álcool*	16.816	58.417

**Table 4:** Day Lag Correlations between New Cases and the keywords in Poland.

	Maseczki	Dezynfekcji	Antybakteryjny
0 day lag	0.38 ***	−0.21 ***	−0.33 ***
1 day lag	0.36 ***	−0.23 ***	−0.34 ***
2 day lag	0.35 ***	−0.25 ***	−0.36 ***
3 day lag	0.33 ***	−0.27 ***	−0.36 ***
4 day lag	0.35 ***	−0.28 ***	−0.37 ***
5 day lag	0.33 ***	−0.29 ***	−0.38 ***
6 day lag	0.33 ***	−0.31 ***	−0.38 ***
7 day lag	0.33 ***	−0.32 ***	−0.39 ***
8 day lag	0.31 ***	−0.33 ***	−0.39 ***
9 day lag	0.30 ***	−0.33 ***	−0.40 ***
10 day lag	0.28 ***	−0.34 ***	−0.40 ***

*** *p*-value < 0.001.

**Table 5:** Day Lag Correlations between New Cases and the keywords in Portugal.

	Máscara Cirúrgica	Desinfetante	Álcool
0 day lag	0.40 ***	0.02 ***	0.15 ***
1 day lag	0.48 ***	−0.01 ***	0.15 ***
2 day lag	0.48 ***	−0.04 ***	0.09 ***
3 day lag	0.51 ***	−0.04 ***	0.04 ***
4 day lag	0.43 ***	−0.06 ***	0.03 ***
5 day lag	0.47 ***	−0.06 ***	0.01 ***
6 day lag	0.50 ***	−0.07 ***	0.04 ***
7 day lag	0.51 ***	−0.07 ***	0.01 ***
8 day lag	0.55 ***	−0.08 ***	0.03 ***
9 day lag	0.58 ***	−0.08 ***	−0.01 ***
10 day lag	0.56 ***	−0.09 ***	−0.02 ***

*** *p*-value < 0.001.
